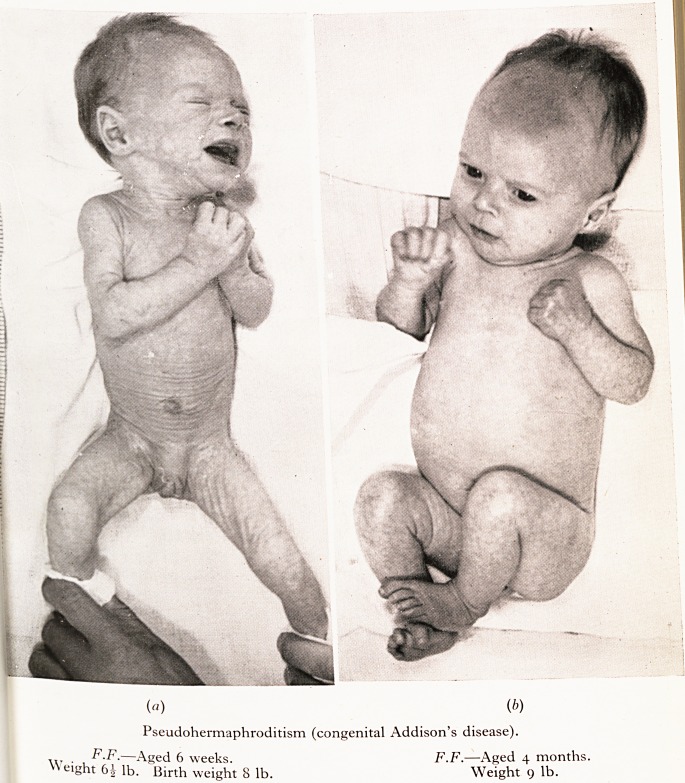# Marasmus

**Published:** 1953

**Authors:** A. V. Neale

**Affiliations:** Professor of Child Health in the University of Bristol.


### "MARASMUS"

BY

A. V. NEALE, M.D., F.R.C.P., D.P.H.
*Professor of Child Health in the University of Bristol.*

The history of human progress is well reflected in the changing views of doctors about wasting in infants, which has at different times been called marasmus, infantile atrophy and athrepsia. We have moved on a little from the days of Hieronymus Mercurialis who discusses in his *De Morbis Puerorum* of 1583 the various ways in which "bewitchment" of the child was alleged to occur and notes two points to establish the diagnosis: (*a*) "if the wasting does not yield to any natural remedies or skill or (*b*) if there is no other cause external or internal in the child (or its nurse)". A diagnosis of "idiopathy" was dangerous!

Great attempts were made to stem the tide of infant mortality. There is evidence in the London Bills of Mortality that in 1730–1749 three-quarters of all children born alive died before they reached the age of five. By the end of the eighteenth century about half the children born in London died before age five. In the early history of the Foundling Hospital in London (founded 1745) there were terrible years, the calamity of 1756 being the worst, when fifteen thousand infants were brought in and, as a result of overcrowding, lack of adequate feeding, and the presence of mortal acute infection, only four thousand four hundred "lived to be apprenticed"! A legal measure was later passed to compel the parishes of London to keep their "parish infants" in country cottages where they lived and throve. Some light is thrown upon at least one factor in the scene of starvation by a verse contained in a hymn which was regularly used in the chapel of the Foundling Hospital, London, about 1774:

> The helpless babe, by hunger prest,
> Clings to the famished mother's breast:
> In vain it ev'ry effort tries;
> Life's fountains yield it no supplies.

Dr. Andrew Combe, writing in 1841, asked if this mortality constituted a necessary part of Divine Providence which man could do nothing to modify, or whether it proceeded chiefly from secondary causes, purposely left under our own control and which we may render innocuous by making ourselves acquainted with the infant constitution and its particular different functions.

Undoubtedly an attitude of fatalism governed the outlook for many more years and in 1881 Henoch continued to deplore the high mortality from wasting disease in the first years of life. He noted that of 7,815 infants under the age of one year, treated at the Royal Charité Hospital, Berlin, over a period of ten years, 5,368 (70 per cent.) failed to survive, and many of the deaths were due to profound wasting on admission. Henoch was pleased to note that Nature has assigned the newborn child to the mother's breast and disregard of this principle in those days was fraught with great dangers. Despite the fact that the phys was often left in " sadness and resignation " he could sometimes hope for su in treating a child even when the fat had steadily disappeared, the skin on the and the whole body had become flabby and wrinkled and the skin brawny desquamation. Henoch noted that even at this stage the functions of the mentary canal, might remain quite normal or almost so; and by suitable nou ment and care " we may still avert the threatened exhaustion and initiat covery ". In the light of our modern knowledge regarding the diverse caus marasmus it is a happy thought that the capable nurse could, even in those tressing times, resuscitate a dying infant simply by feeding it!

That there was some accurate knowledge about the subject is illustrated by story of congenital pyloric stenosis. Dr. Patrick Blair (1717) observed child was five months old and was so emaciated that he appeared rather to decreased since the time of his birth, the whole body not weighing abov pounds . . . the coats of the ventriculus were thick and the pylorus was al cartilaginous so that no nourishment could have passed into the intestin Although Dr. Christopher Weber again described the condition in 1758, Dr. Michael Underwood in 1784, these were rare occasions and no distin causes of " infantile atrophy " were discussed. Time passed on, and L Parsons in the *Goulstonian Lectures* of 1924 on " Wasting Disorders in Infancy " observed that the subject appeared to be lacking in general inte and was by many still regarded as threadbare despite the persistence of a death-rate from these disorders. No doubt, as Parsons said, infantile atro was principally a " social disease ", infection being a common factor as shown by its relative rarity in babies completely breast fed. Vicious circle metabolic disturbance were recognized and stages of decline were reache which, paradoxically, the basal metabolism increased with progressive emacia and dehydration, and in which poor retention of mineral salts and nitro would upset the whole physiological balance.

Thirty years ago a great deal of discussion occurred about the possibili pylorospasm, but while much of it was fruitless it did lead to a more gen recognition of true pyloric stenosis, mainly through the work of John Thom of Edinburgh and Frederick Still of London. This advance in diagnosis and skill acquired by surgeons in performing the Rammstedt operation of p otomy led to a recovery rate of 50 per cent. in 1924. These figures have very greatly improved since. However, there was at the same time muc confirm the older view of Dr. Charles West that the onset of infantile atro was often due to the supply of insufficient and unsuitable food rather than primary disorder of digestion and absorption. " Intestinal indigestion " then, as it is occasionally even now, used as a nebulous label to cover inadeq diagnosis.

Considerable advances have been made in the methods of investigatio cases of marasmus and in their precise diagnosis and it is now only rarely there is a failure to determine the facts. It must, however, be remembered underfeeding is still by no means uncommon and that sometimes, even children may suffer from it in an extreme degree. Air swallowing and secon projectile-like vomiting may contribute to the starvation and finally an apat state with deprivation anorexia may cause death. My first example is that child who seemed to be on the point of death but who was really suffering from starvation like that seen in countries devastated by war.

D.P. was admitted at the age of ten months in the state depicted in the photograph. Prolonged underfeeding, with intermittent secondary aerophagic colic and vomiting reflected an unusual degree of low-grade mothercraft. The gross marasmus with feeble cry and dehydration, apathy and stupor suggested that death was near. No organic disease could be found and there was a wonderful response to a carefully adjusted dietary. Finally the child became quite normal in appearance and behaviour.

A simple laxity of the cardiac sphincter is frequently the reason for habitual vomiting or easy regurgitation especially during the period of milk feeding. There may be considerable difficulty in maintaining caloric balance. Fortunately thickening of feeds and placing the baby in the inclined position with the head raised is often effective. The early introduction of cereal feeding may cause rapid improvement but, in addition, some tricks of nursing may be needed.

B.K.W. whose birth-weight was 7½ lb. was a hungry, restless and rather dehydrated baby of only 10 lb. at four months. Milk feeds were regurgitated from time to time and there was no gain in weight. Addition of a wheal cereal cured the symptoms and the weight went up rapidly to 13½ lb.

Herniation of the stomach at the diaphragmatic hiatus may quickly produce infantile dysphagia, vomiting which may be severe and protracted or appear in short disabling bouts, and " blood in vomit ". Haematemesis in infancy from cardio-oesophageal ulceration is usually due to a hiatus hernia.

A.D. had vomited from the earliest weeks of life and at the age of two months was only a few ounces above his birth-weight of 7 lb. He was hungry and lively. Persistent underfeeding with cows' milk was suspected, but careful observation showed blood streaks and debris in the vomited acid material. A small hiatus hernia was found. By suitable regulation of feeding and the addition of a cereal and propping the baby up after feeds, this condition subsided (clinically and radiologically) and after some months' careful supervision the growth, body weight and general development were normal.

All sorts of persistent infection will seriously interrupt a baby's progress. Congenital syphilis may, however, occur in a well-nourished baby. Congenital tuberculosis (hepato-pulmonary) may be suspected on circumstantial and clinical evidence. Persistent respiratory sepsis in and following the neonatal period may make a child seriously ill with anorexia, fever, metabolic wasting, secondary alimentary irritability, toxic diarrhoea with fermenting stools and excoriation of the perineal skin.

R.T., one of twins, had pertussis and pneumonia at two months of age. His previous good progress stopped and gradually he drifted to below his birth-weight. The respiratory sepsis, upper zone infection and lung collapse were very troublesome. With good nursing and treatment with aureomycin he recovered slowly and is now normal with a weight of 14 lb.

The irritable irritated baby with eczema is a well-known " waster ". The peculiar difficulties in feeding and nursing may soon set up an intractable duel between mother and baby, and both may break down—the mother in tears and the baby in weight. The condition is common and is full of interest as a social problem, for the psychological strains within the family are often an important contributory cause.

Inherited pancreatic fibro-cystic disease has, unfortunately, become common in paediatric practice. Early failure to thrive and to gain weight in spite of an apparently normal appetite and food intake are suspicious symptoms. Th[illegible] existence of bronchial infection, and foetid greasy stools with a low or a[illegible] intestinal trypsin, confirm the diagnosis.

**S.D.** failed to thrive since normal birth. There was troublesome cough and per[illegible] bronchitis. At four months of age he had only advanced from birth-weight of 7$\frac{1}{4}$ [illegible] 8$\frac{1}{4}$ lb., despite the fact that he was persistently hungry and took feeds well. The[illegible] were pale and offensive. His duodenal fluid contained normal bile but a very low [illegible] activity. It is noticeable in his photograph that despite the " wasting " (failure to [illegible] he is alert and is trying heroically to counteract the intestinal indigestion which is d[illegible] lack of the pancreatic enzymes.

Whilst the diagnosis of coeliac disease is less frequently delayed than for[illegible] an advanced stage of wasting may be seen.

**D.C.** was normal at birth and weighed 8$\frac{1}{2}$ lb. She was not breast fed and her [illegible] declined in the later months of infancy and although the detailed history is no[illegible] observation at twenty-three months indicated severe wasting associated with an[illegible] abdominal distension and grave weakness. One large pale offensive stool was passed[illegible] The diagnosis was clearly coeliac disorder which had drifted on probably since [illegible] twelve months of age. Eight grams of fat were excreted in the stools daily. The[illegible] normal tryptic activity of the duodenal fluid. Treatment has been very effective a[illegible] favourable progress is shown in the photograph.

A long list of detectable causes of wasting disease in infancy could be [illegible] Some are self-evident, such as habitual vomiting; oral and oesophageal [illegible] infection; upper respiratory sepsis with perhaps degrees of snuffling nas[illegible] struction; feeding difficulty in cleft palate or in micrognathus; episodes o[illegible] longed anorexia in general diseases, such as acrodynia (pink disease), spa[illegible] mentally deficient infants; recurrent vomiting in chronic subdural haem[illegible] or some forms of congenital heart disease with cardiomegaly; persistent vo[illegible] in pertussis or even in irritative nasopharyngitis may lead to profound wa[illegible] renal insufficiency associated with congenital abnormalities may even[illegible] present as a general wasting syndrome. Endocrine deficiency disorder[illegible] greatly inhibit growth and body weight in infancy and some remarkably [illegible] esting examples occur, for instance the pituitary gland may be compressed[illegible] parahypophyseal congenital cyst so producing an infantile form of at[illegible] Simmond's pituitary cachexia. Inflammation, injuries or neoplasm [illegible] mid-brain may disturb the normal neuropituitary mechanisms and lead to s[illegible] effects. The infant cretin, whilst not usually showing severe wasting[illegible] appear to be wasted because of its dry skin and wrinkled appearance.

Metabolic derangements leading to wasting in infancy are essentially [illegible] manifestations of tissue enzyme defect. A wasted baby with a very large [illegible] liver may be so affected because too much of its glucose is being convert[illegible] fixed as glycogen (von Gierke syndrome); or a baby may similarly d[illegible] hepatomegaly with steatosis. In both these conditions there is an unba[illegible] one-way traffic of enzyme and chemical exchange. Galactosaemia with g[illegible] suria is a perfect example of metabolic enzyme error: milk feeding is not to[illegible] in this disorder because the lactose is metabolically toxic and leads to rap[illegible] enlargement, haemorrhagic tendency and acute wasting. A quick decis[illegible] feed the baby on a lactose-free preparation will save life (glucose can be [illegible] bolized normally) and later a gradual lactose balance is achieved.

The antidiuretic hormone is of low potency in infancy and body-water [illegible]

ne baby's body is 85 per cent. water) is unstable. Sometimes the infant's renal bular structure, which is functionally immature, varies in its reaction to anti-uretic influences, and wasting with polyuria and dehydration may call for ecial considerations of fluid intake until the renal mechanisms for water con-rvation are established. Renal tubular re-absorption thresholds may be regular for phosphate, chloride, glucose, bicarbonate, or sodium: in fact one more of these substances may be excessively lost in the urine and thus produce, infancy, clinical syndromes of wasting with anorexia. Simple renal glycosuria uses no upset but it may be accompanied by urinary excretion of amino-acids hich sets up a train of general tissue disturbance with wasting and later bone anges (Fanconi Syndrome). " Renal acidosis " due to persistent excess loss base may be suspected when the urine (in fresh specimens) is consistently kaline in a " marasmic " baby. A recent advance in these metabolic studies s been the recognition that a baby may show wasting, failure to grow, variable iorexia with periodic vomiting, and a peculiar severe constipation, with no normal finding but persistent hypercalcaemia.

T.L., whose birth-weight was 5½ lb., has a normal sibling and his parents are healthy. is progress was such that he reached 7 lb. 11 oz. at ten weeks. After this, however, he oved up slowly and at thirteen months he weighed 12 lb. Severe constipation with hard ools, a capricious appetite and a refusal to tolerate mixed diet was henceforth associated ith episodes of vomiting and even over several months there was no gain in weight. He as cheerful and playful, but would not gain an ounce! Yet the only abnormality was rsistent (idiopathic) hypercalcaemia—the serum Ca varying between 17 and 18 mg. per nt. We have been unable to lower the Ca after three months without vitamin D, and ere is nothing to suggest parathyroid disease. The diagnosis of this case of " wasting " so far a metabolic mystery.

This case is not unique, several others have come to light since we have tested the serum lcium level in suspect cases.

The term " pseudo-pyloric stenosis " crept into the literature some years ago. vere projectile vomiting with wasting but no visible gastric peristalsis or pal-ble pyloric tumour was confusing until it was noted that these babies all had ngenital adrenal cortical deficiency (infantile Addison's disease), and strikingly, e female infants so affected showed a pseudo-hermaphrodite appearance.

F.F., a full term female 8-lb. baby, after a week of restful breast-feeding, vomited all eds and " pumped it up ". Severe wasting was manifest at one month. The external ns of pseudo-hermaphroditism and a great decline of plasma chloride decided the agnosis of congenital adrenal cortex insufficiency (chiefly a poor production of the ineralocorticoid fraction). Desoxycorticosterone acetate (D.O.C.A.) may quickly remedy e syndrome and, as in this case, stop the vomiting and enable a normal salt balance to maintained with normal feeding.

In all cases of wasting in infancy the possibility of a structural abnormality hich needs surgical treatment must be considered. Examples are oesophageal enosis (oesophageal atresia must be recognized in the first day of life), oeso-hageal hiatus herniation, or even the rare condition of circular compression om a double aortic ring embracing the tracheo-oesophageal structure in the perior mediastinum. Diaphragmatic herniation, usually through the left dia-hragm, may present as a wasting disorder associated with bouts of obstructive miting and severe feeding difficulty. Malrotation of the mid-gut may be cognized early by the special character of the bilious vomiting due to the con-nital stricture at or near the duodeno-jejunal zone. Congenital mesenteric cyst (due to a local duplication of the intestine) may be recognized. Occasio[illegible] the infantile form of Hirschsprung's disease with visible colonic peristal[illegible] observed. Recurrent or intermittent intussusception may be detected. Del[illegible] effects of birth injury and particularly the presence of subdural haemat[illegible] causing vomiting and wasting in association with the increased intracranial [illegible] sure as indicated by bulging anterior fontanelle and an unusual rate of circ[illegible] ferential head enlargement, demands urgent treatment.

There is no doubt that quick and accurate diagnosis of the cause of wastin[illegible] an infant is imperative. A baby's reserves are limited and if nothing is don[illegible] condition may deteriorate very rapidly.

I am grateful to my colleagues and to the nursing staff in the Bristol R[illegible] Hospital for Sick Children for their general help in this paper and to the Un[illegible] sity Department of Clinical Photography for the photographs.

### REFERENCES

Hieronymus Mercurialis (Jerome Mercuriale): *De Morbis Puerorum*, Vienna, 15[illegible]

Blair, P.: *Philosoph. Trans. Roy. Soc.*, 1717, **353**, 631.

Weber, C.: *Mors ex spasmo pylori*, Thesis, Univ. of Göttingen, 1758.

Underwood, M.: *Diseases of Children*. London, 1784.

Combe, A.: *Management of Infancy*, Edinburgh, 1841.

West, C.: *Lectures on Diseases of Children*, London, 1848.

Henoch, E.: *Children's Diseases*, Berlin, 1881, and New Sydenham Society, Vol[illegible] 1889.

Thomson, J.: *Treatment of Sick Children*, Edinburgh, 1898.

Still, G. F.: *Trans Path. Soc. Lond.*, 1898–9, **50**, 86.

Parsons, L. G.: *Lancet*, 1924, **1**, 793 and 939.

PLATE I

(*a*) Malnutrition. (*b*)

*D.P.*—Aged 6 months. Weight $6\frac{1}{2}$ lb.

*D.P.*—Aged 7 months. Weight $7\frac{1}{2}$ lb.

PLATE I

(*c*)

*D.P.*—Aged 8 months. Weight 9 lb.

(*d*)

*D.P.*—Aged 10 months. Weight [illegible]

PLATE II

*B.K.W.*—Wasting due to simple oesophageal regurgitation.
Aged 6 months.

PLATE III

(*a*) Oesophageal Hiatus Hernia. (*b*)

*A.D.*—Aged 2 months. Weight $7\frac{1}{2}$ lb. *A.D.*—Aged 19 months. Weight 30[illegible]

PLATE IV

(a) (b)

Wasting due to persistent chest infection.

*R.T.*—Aged 4 months. Weight 6 lb. *R.T.*—Aged 6 months. Weight 14 lbs.

PLATE V

(*a*) *S.D.*—Note the acute devitalizing effect of the relapsing respiratory infection.

(*b*) *S.D.*—Aged 4 months. Weight $8\frac{1}{4}$ lb.

(*c*) *S.D.*—Aged 4 months. Weight $8\frac{1}{4}$ lb.

(*a*) Coeliac Disease. (*b*)

*D.C.*—Aged $2\frac{1}{2}$ years. Weight 17 lb.

*D.C.*—Aged $3\frac{1}{2}$ years. Weight 31 lb.

PLATE VII

Idiopathic hypercalcaemia.
*T.L.*—Aged 13 months. Weight 12 lb.

PLATE VIII

(*a*) (*b*)

Pseudohermaphroditism (congenital Addison's disease).

*F.F.*—Aged 6 weeks.

Weight $6\frac{1}{2}$ lb. Birth weight 8 lb.

*F.F.*—Aged 4 months.
Weight 9 lb.

## Figures and Tables

**(a) (b) f1:**
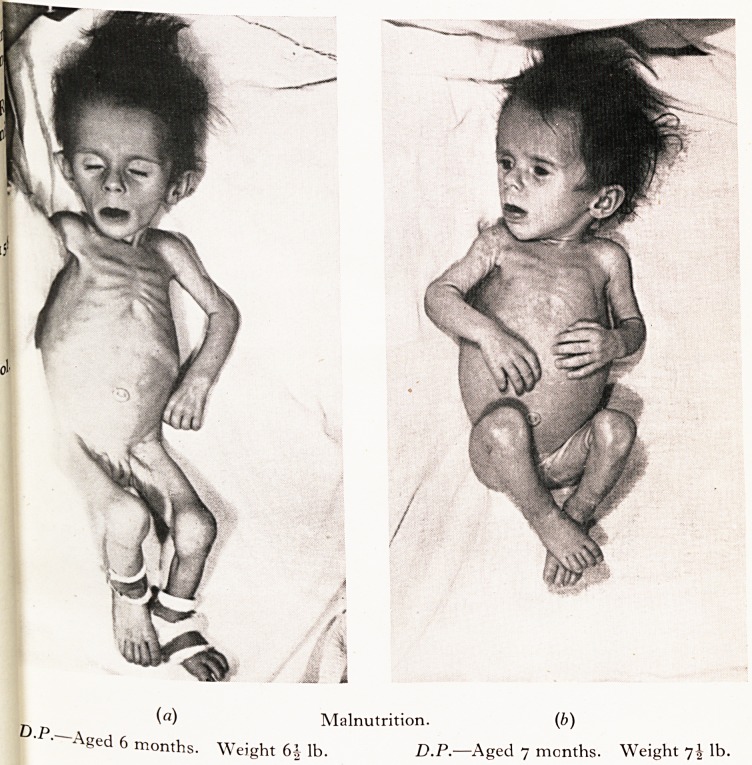


**(c) f2:**
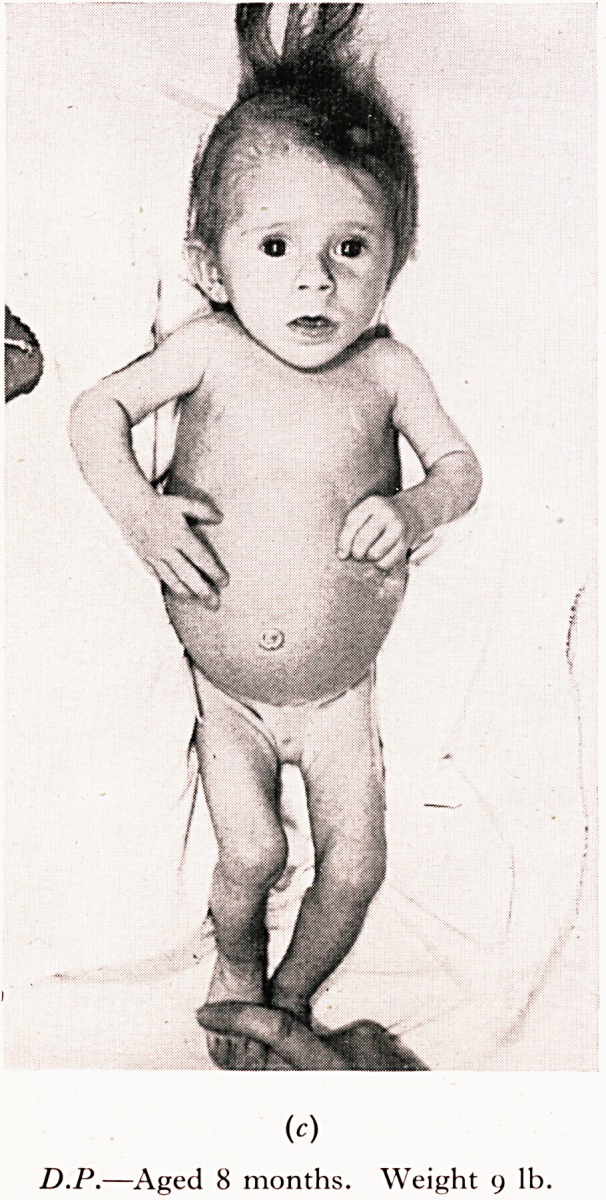


**(d) f3:**
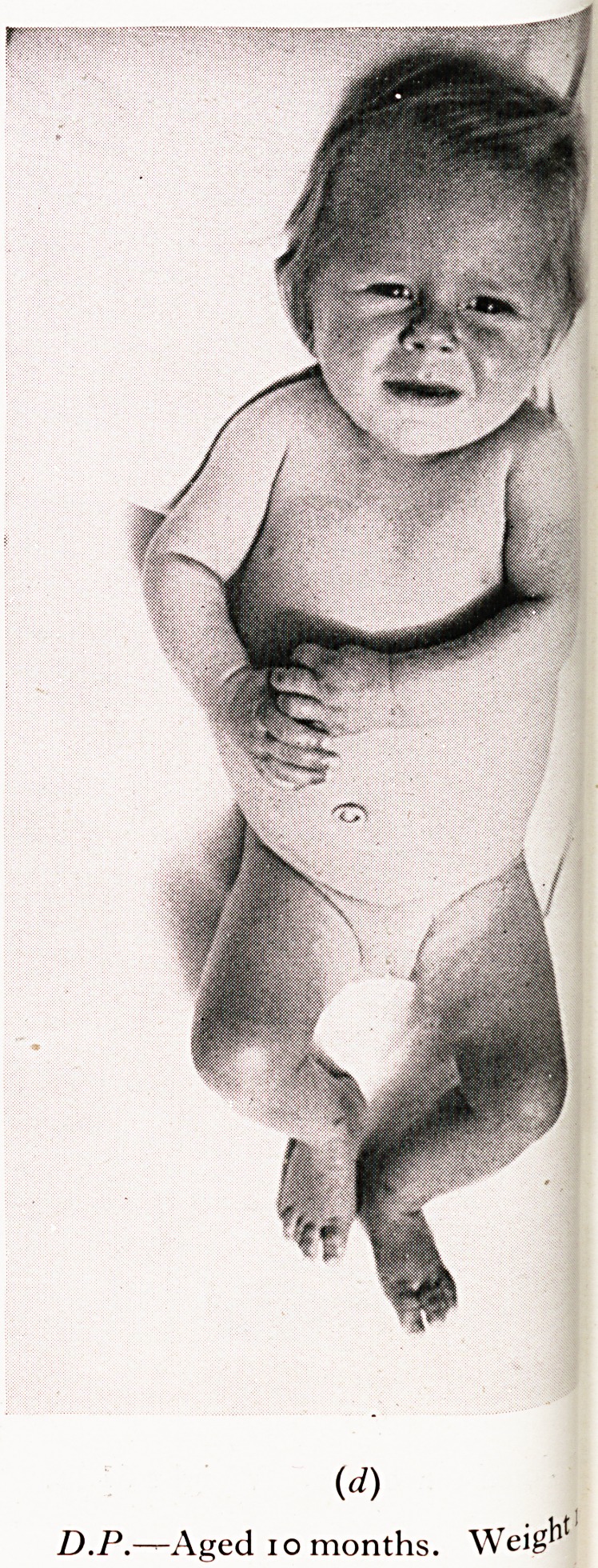


**Figure f4:**
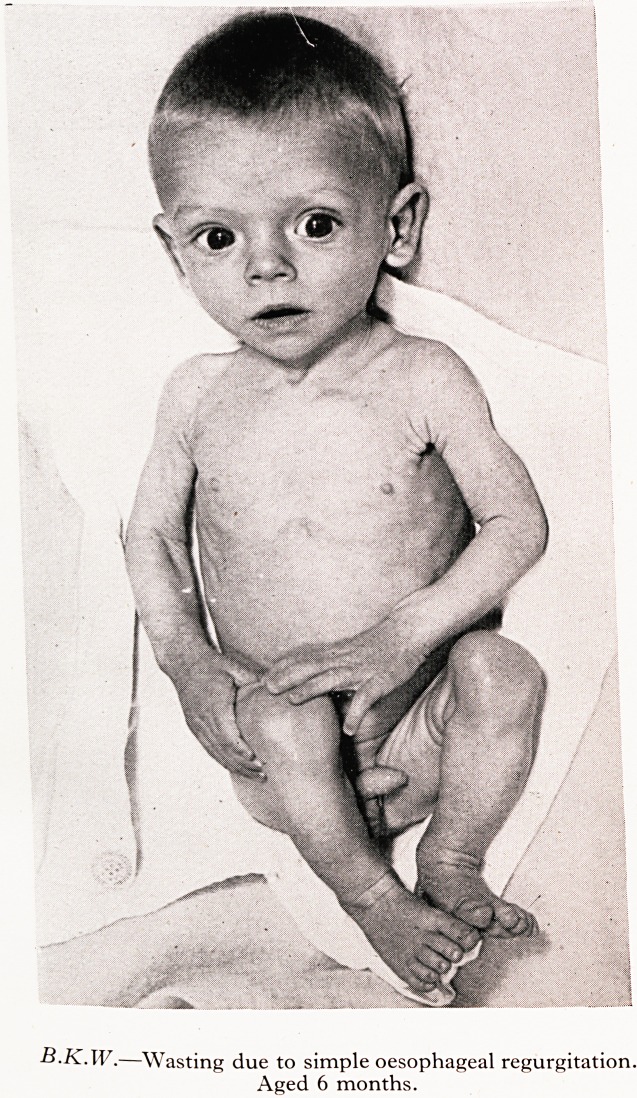


**(a) (b) f5:**
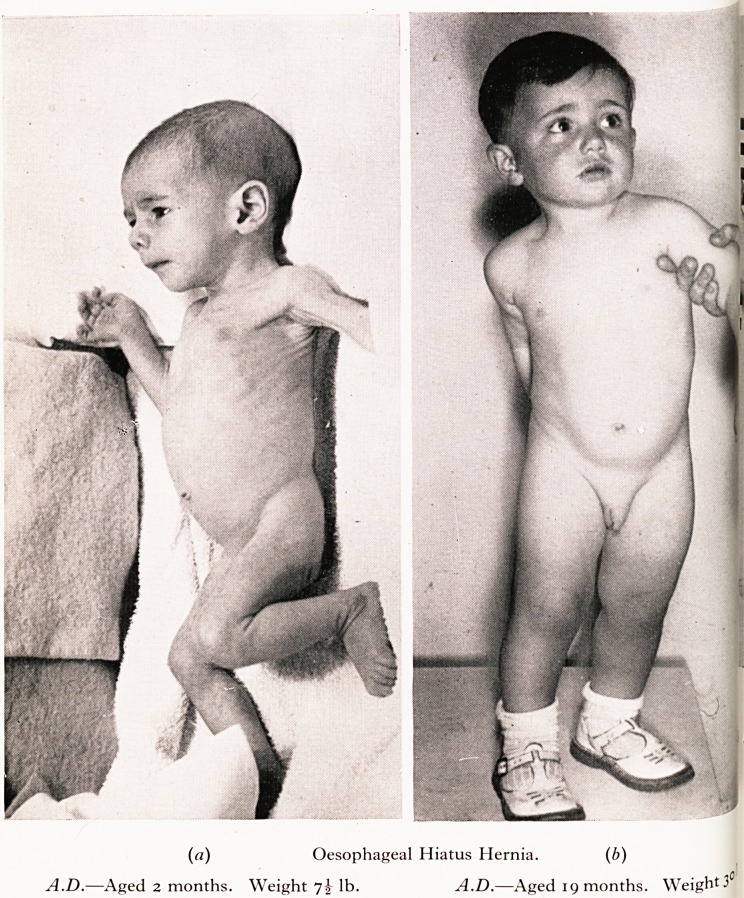


**(a) (b) f6:**
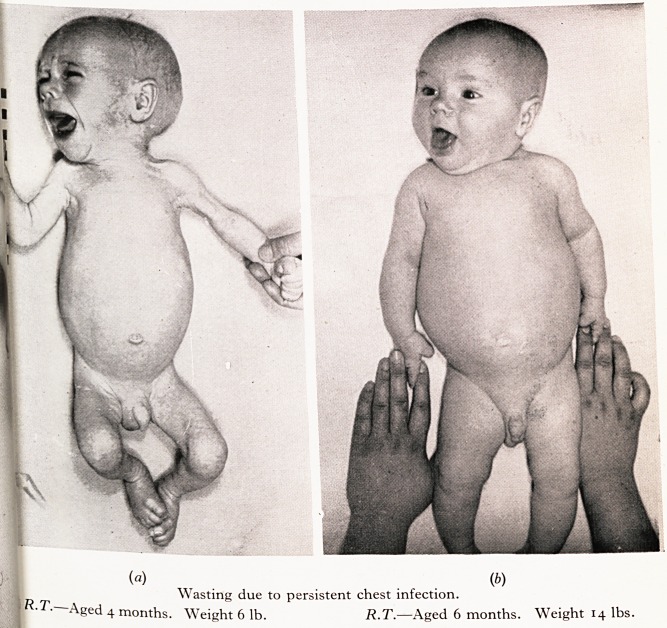


**(a) f7:**
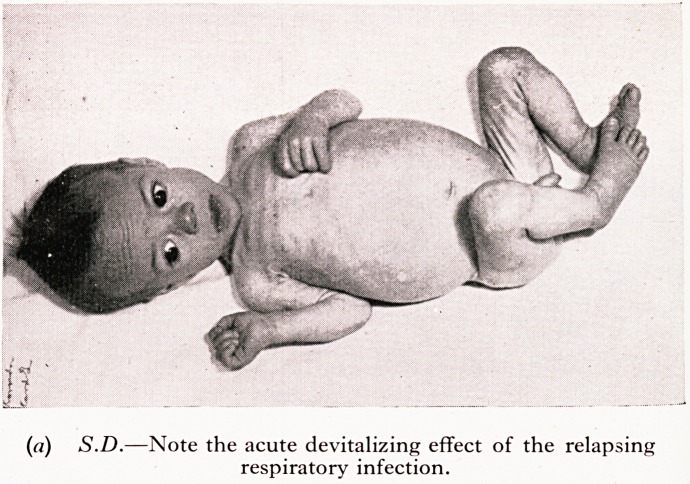


**(b) f8:**
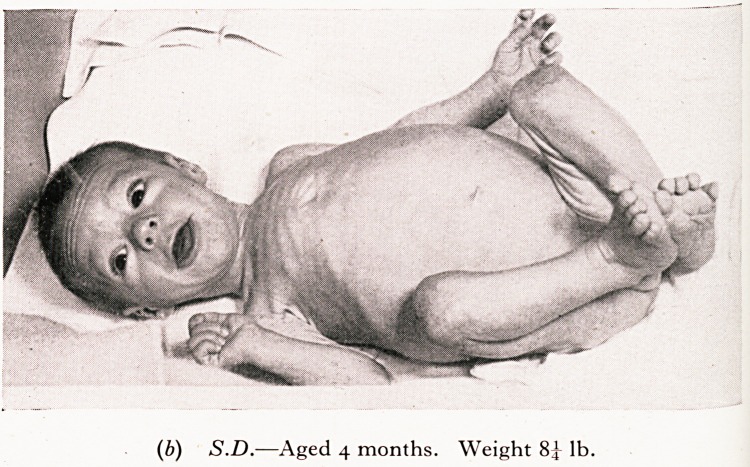


**(c) f9:**
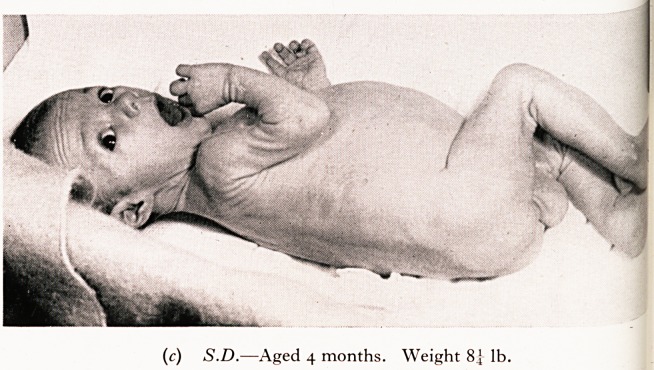


**(a) (b) f10:**
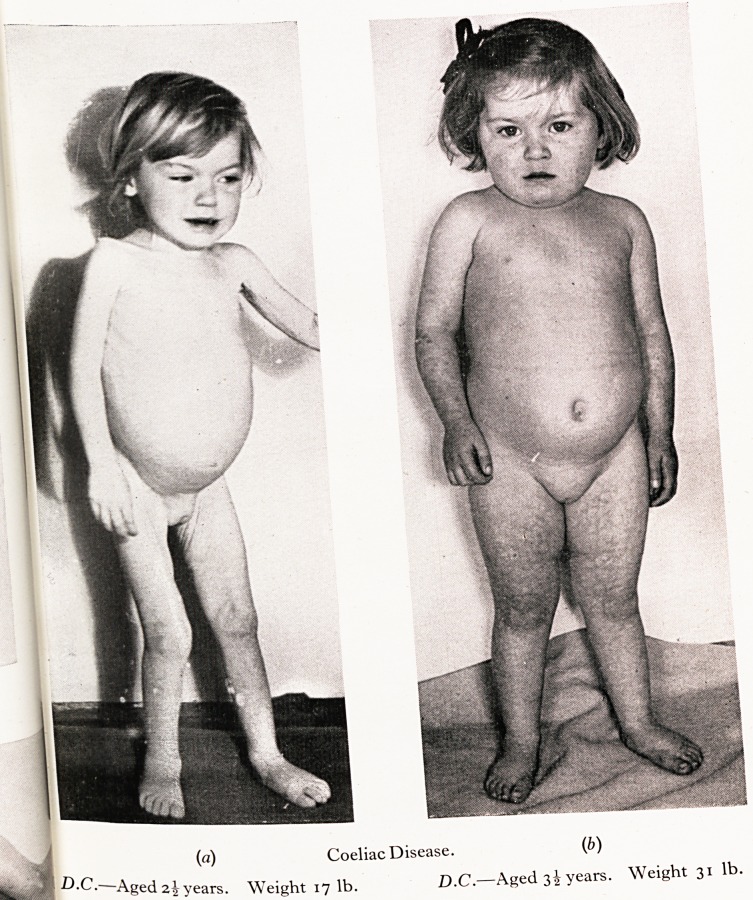


**Figure f11:**
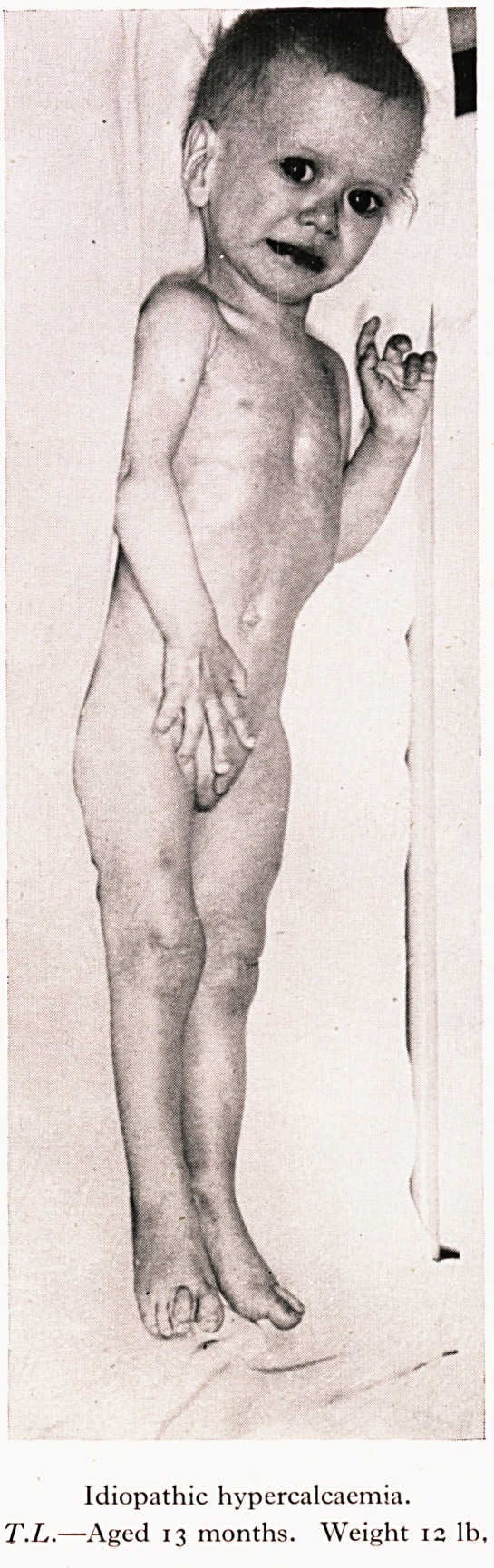


**(a) (b) f12:**